# Antioxidant Capacity and Thermal Stability of *Arthrospira platensis* Extract Encapsulated in Starch Sodium Octenyl Succinate with Freeze-, Spray-, and Nanospray-Drying

**DOI:** 10.3390/molecules30061303

**Published:** 2025-03-13

**Authors:** Vesta Navikaitė-Šnipaitienė, Dovilė Liudvinavičiūtė, Ramunė Rutkaitė, Vaida Kitrytė-Syrpa, Michail Syrpas

**Affiliations:** 1 Department of Polymer Chemistry and Technology, Kaunas University of Technology, Radvilenu Rd. 19, LT-50254 Kaunas, Lithuania; vesta.navikaite@ktu.lt (V.N.-Š.); dovile.liudvinaviciute@ktu.lt (D.L.); 2 Department of Food Science and Technology, Kaunas University of Technology, Radvilenu Rd. 19, LT-50254 Kaunas, Lithuania; vaida.kitryte@ktu.lt

**Keywords:** *A. platensis*, phycocyanin, encapsulation, antioxidant, thermal stability

## Abstract

*Arthrospira platensis* is a filamentous cyanobacterium produced commercially for human consumption, and it is a source of phycocyanin (PC), which recently stirred up great interest due to its anti-inflammatory, radical scavenging, antioxidant and hepato-protective properties. This work has studied the encapsulation of *A. platensis* extract in starch sodium octenyl succinate by employing freeze-drying and two spray-drying techniques, conventional and nanospray-drying. The main characteristics and properties, including PC encapsulation efficiency, size, colour, and thermal stability of the capsules, were evaluated. Moreover, the antioxidant capacity of encapsulated extract and release of PCs into saliva simulant, were studied and compared. Similar PC encapsulation efficiency was achieved using freeze-drying and nanospray-drying techniques with values of 67–71% and 70–78%, respectively. Meanwhile, the conventional spray-drying method achieved significantly lower encapsulation efficiency values (38–42%). The thermal stability of encapsulated *A. platensis* extract was improved as demonstrated by the higher decomposition temperature, which was increased by 8–11 °C, 11–15 °C, and 22–23 °C for spray-dried, nanospray-dried and freeze-dried samples, respectively. The nanospray-drying technique allowed the production of the smallest particles with an average diameter of 2–14 µm, good colour and thermal stability, and antioxidant capacity. Overall, the results demonstrated the potential of *A. platensis* extract encapsulation in modified starch using several techniques with potential application as bioactive ingredients in nutraceutical or pharmaceutical products.

## 1. Introduction

Over the last few years, microalgae have gained interest as an emerging source of functional ingredients, as they represent high levels of proteins, carbohydrates, phenolic compounds, vitamins, and minerals [1,2]. *Arthrospira platensis* (commonly known as Spirulina) has received significant attention among microalgae due to its high protein level, reaching 60–70% and bioactive metabolite content, providing health-enhancing properties [3]. The light-harvesting complexes of *A. platensis*, namely phycobiliproteins such as phycocyanin (PC) and allophycocyanin, can be extracted as water-soluble blue colour pigments [4]. PC has especially attracted interest due to the broad spectrum of reported activities, including antioxidant, anti-inflammatory, and immunomodulatory activities [5,6,7].

Previous studies have demonstrated the possible advantages of consistent spirulina consumption in preventing various clinical conditions linked to inflammation and oxidative stress [8]. Such features could be successfully exploited in the treatment of oral cavity tissues. For example, in dentistry, spirulina was assessed for its oxidative properties in the healing of oral submucous fibrosis [9]. Furthermore, it was found that spirulina mouthwash was effective in reducing dental plaque and gingivitis [10]. Meanwhile, bacterial biofilms in the gingival edge cause periodontitis, an inflammatory condition in which indigenous gram-negative periodontal bacteria trigger a cascade of inflammatory reactions in the periodontal tissues, with reports indicating that the antioxidant properties of spirulina or its extracts can positively influence treatment procedures of periodontitis [11,12,13]. As the primary objectives of therapy for patients with chronic periodontitis are to halt disease progression and to resolve inflammation, the local application of the remedy into the periodontal pocket could be very advantageous, both in terms of rising the concentration directly in the action site and in preventing systemic side effects such as gastrointestinal complaints, depression, and tachycardia when using conventional treatment methods by oral administration. As *A. platensis* appears to be a promising agent with a wide array of antibacterial, antioxidant, anti-inflammatory, and anti-fungal properties with low toxicity and minimal side effects, its controlled delivery within periodontal pockets could alter the pathogenic flora and improve clinical signs of periodontitis. However, little is known regarding spirulina extracts’ stability, release, and antioxidant properties in salivary substitutes.

Regardless of their potential application, PC-rich extracts are known to be unstable due to the protein structure and are affected by many factors, such as light, pH, and temperature [14]. Hence, much effort has been put into developing strategies to stabilize this valuable pigment. Encapsulation is a technique by which sensitive compounds can be protected from degradation through a wall material, which controls the release and could improve the bioavailability of bioactive compounds [15]. However, especially for food, nutraceutical, and pharmaceutical applications, the wall material must be food-grade, biocompatible, and biodegradable [16]. Moreover, it should protect against external conditions and processing factors, such as the negative impacts of temperature, pH, light, and oxygen, thus enhancing stability and prolonging shelf life [17]. In response to these demands, various techniques have been suggested to produce PC micro- or nano-capsules, such as spray-drying, freeze-drying, extrusion, electrospraying, and electrospinning methods [18,19]. Spray-drying and freeze-drying are techniques that have been successfully applied in pharmaceutical technology for the production of dry powder for bioactive compounds delivery systems. However, nanospray-drying presents several advantages over the conventional spray-drying and freeze-drying methods. Nanospray-drying improves powder formulation and release of active ingredients into the medium of interest. Conventional spray-drying typically yields larger particles, which may prove less efficacious for certain applications, especially where higher bioavailability is needed [20]. Additionally, the operation of spray-drying at quite high temperatures may further contribute to the degradation of heat-sensitive compounds, thereby reducing encapsulation efficiency. Despite freeze-drying operating at low temperatures, thus preserving bioactive compounds, the obtained particle might be quite large and less uniform than those produced by nanospray-drying [21] and therefore less suited for immobilisation in thin periodontal films. In comparison other prevalent and efficient encapsulation techniques, such as emulsification or preparation of liposomes involving creation of liquid-based formulations, may not ensure the same level of long-term stability or protection against environmental factors as those in powdered form.

Wall materials like modified starch, maltodextrin, gum arabic, whey protein isolate, alginate, and carrageenan have been used to encapsulate *A. platensis-*derived PC and extracts [22]. For instance, *A. platensis*-derived PC was encapsulated within amorphous chains of cross-linked starches of various origins by using the freeze-drying method, and studies confirmed the feasibility of using starch-based systems as wall material for extending-release pharmaceutical formulations [23]. Furthermore, maltodextrin and gum arabic were selected for PC encapsulation, and studies demonstrated that freeze-dried microencapsulated PC possessed good antioxidant properties, pigment stability, and resistance to high temperatures [24]. Additionally, sodium dodecyl sulfate-based surfactant micelles have shown protective effects against PC degradation [13]. In another study, using spray drying and different combinations and ratios of wall materials such as maltodextrin, gum arabic, whey protein isolate, and sodium caseinate to protect PC, the highest microencapsulation efficiency was obtained with whey protein isolate and gum arabic as wall material [25]. In addition, a study comparing several techniques, including freeze-drying, spray-drying, and ball-milling, was carried out for the encapsulation of *A. platensis* using trehalose and trehalose-maltodextrin mixtures. The study demonstrated that the highest carrying capacity with a residual amount of PC > 89% after encapsulation was achieved using the freeze-drying method, while the use of ball co-milling for encapsulation caused a complete degradation of the core compound [26]. However, there are no data on the utilisation of starch sodium octenyl succinate as a wall material for the encapsulation of *A. platensis* extract or its bioactive metabolites to date, although starch sodium octenyl succinate is known as a safe food additive and a perfect wall material for the encapsulation of various bioactives, including volatiles [27].

Furthermore, there is a lack of information on the influence of different techniques used for the encapsulation process and comparing the characteristics and properties of the obtained materials. For example, the formulations of bioactives to be further used in thermal processing would require increased thermal stability. This will be very important when the encapsulated PC extracts are further applied in the formation of periodontal films, which eventually are prepared by various techniques involving heat treatment such as solvent casting, semisolid casting, hot melt extrusion, solid dispersion extrusion, rolling, and other methods.

This work examined the impact of the various encapsulation techniques on the encapsulation efficiency of *A. platensis* extract, the morphology of the particles, colour and thermal stability, antioxidant activities, and release of PCs from the capsules into saliva simulant. Different techniques, such as freeze-drying and two spray-drying techniques, conventional and nanospray-drying, were employed for *A. platensis* encapsulation in starch sodium octenyl succinate particles. This study aimed to compare the characteristics and thermal properties of the encapsulated extract to suggest the optimal method for encapsulating *A. platensis* extracts to be regarded as potential bioactive ingredients for nutraceutical or pharmaceutical applications.

## 2. Results and Discussion

### 2.1. Encapsulation of A. platensis Extract in Starch Sodium Octenyl Succinate

Spirulina is an excellent source of nutrients and is widely used because of its unique combination of high-quality proteins, fatty acids, antioxidants, vitamins, and minerals [28]. In this work, *A. platensis* (APE) extract was obtained using the freezing–thawing method. The compositional profile of obtained APE (expressed as g/100 g of dry extract) was protein: 62.4 ± 0.2, carbohydrates: 25.5 ± 0.4, ash: 10.5 ± 0.3, and lipid: 1.6 ± 0.4. Moreover, the amino acid profile of APE was analysed chromatographically. The amino acid profile expressed as % of the protein fraction was as follows: alanine 10.9%, arginine 11.5%, aspartic acid 4.0%, cystine 0.9%, glutamic acid 32.9%, isoleucine 0.7%, leucine 9.6%, lysine 4.6%, methionine 0.7%, phenylalanine 9.2%, proline 0.2%, serine 3.8%, threonine 1.0%, tryptophan 1.3%, and valine 1.7%. Encapsulation effectively protects bioactive compounds from inappropriate environmental conditions and ensures their stability and targeted release [18]. In the first part of this study, the encapsulation of *A. platensis* extract in starch sodium octenyl succinate (SSOS) has been investigated by employing freeze-drying and spray-drying techniques, which are well-known to be suitable for the production of solid microparticles and nanoparticles with encapsulated bioactive components [16]. Firstly, the aqueous formulations consisting of 10% (*w*/*w*) starch sodium octenyl succinate and 1, 2, 3, and 5% (*w*/*w*) of *A. platensis* extract were prepared by using the rotor–stator homogenisation method. The main characteristics of the prepared dispersions, such as component ratio, particle size, and polydispersity index, were determined and are provided in Table 1. The particle size of the formulations varied from 396 to 542 nm by changing the amount of *A. platensis* extract from 1 to 5%. As can be seen from the obtained data, the particle size of the dispersion depended on the amount of the extract in the dispersion and was slightly decreased with the increasing portion of the spirulina. The polydispersity index (PI) values were closer to zero and indicated a homogeneous distribution of dispersion particles.

In the next step, the prepared dispersions were processed using freeze-drying and two spray-drying techniques (Table 2). In total, 12 samples containing 4 different concentrations of encapsulated *A. platensis* extract were prepared by employing 3 techniques to produce 3 groups of the samples: freeze-dried samples (F), spray-dried samples (S), and nanospray-dried samples (NS). As can be seen from Table 2, the total content of PCs depended on the chosen encapsulation technique and the concentration of the extract in primary formulations. The PC content in freeze-dried and nanospray-dried samples varied from 4.3 to 16.6 mg/g and 4.5 to 18.3 mg/g, respectively. Meanwhile, PC content (2.4–9.7 mg/g) detected in spray-dried samples was much lower than the latter techniques, indicating that this particular technique is less favourable for PC encapsulation. In a previous study, when various combinations and ratios of wall materials, including maltodextrins, gum arabic, sodium caseinate, and whey protein isolate, were used for PC microencapsulation, the highest value of total PC content was achieved at 2.03 mg/g [25]. Therefore, the techniques and wall material selected for our study provided significantly higher content of encapsulated PC in solid particles.

In this study, PC’s encapsulation efficiency (EE) was expressed by the percentage of PC obtained in final solid particles compared to the initial preparations. The EE values of PC for various samples are presented in Table 2. As can be seen, the encapsulation technique had the highest impact on encapsulation results. In addition, no significant changes were found in the different content of *A. platensis* extract used in the formulations. The encapsulation efficiency values for freeze-dried and nanospray-dried samples were similar and were varying from 67 to 71% and 70 to 78%, respectively. Meanwhile, EE values for spray-dried samples varied between 38–42% and were almost twice as low as other techniques. Many factors, including carrier, core material, and encapsulation techniques, can influence encapsulation efficiency. Despite the chemical materials used for the encapsulation process, the encapsulation efficiency (EE) depends on many technological factors, such as process technique and duration, temperature, and other parameters. In our case, when the encapsulation process was performed with the spray-drying equipment, a reasonably elevated temperature of 190 °C was used, which caused the reduction in PC due to the degradation of protein structure [29].

**Figure 1:**
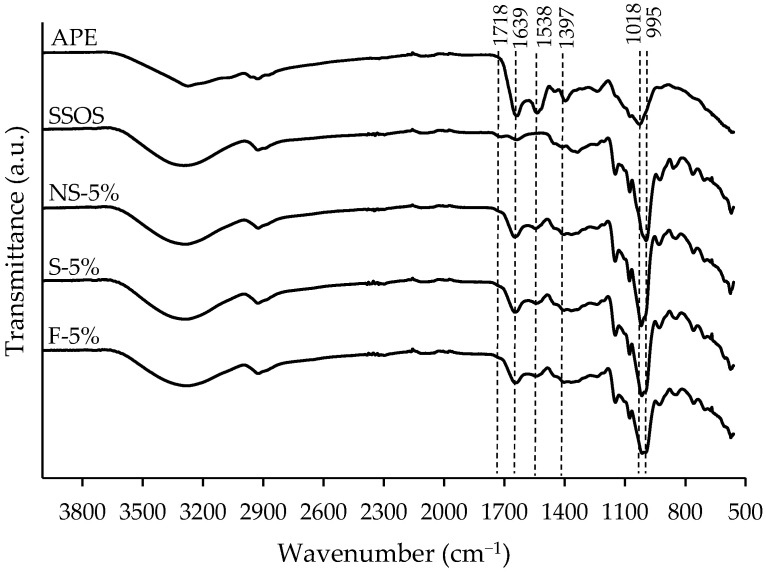
FT-IR spectra of *A. platensis* extract (APE), starch sodium octenyl succinate (SSOS), freeze-dried sample (F-5%), spray-dried sample (S-5%), and nanospray-dried sample (NS-5%).

In the next step Fourier Transform Infrared Spectroscopy (FT-IR) analysis was performed on the powders of F-5%, S-5%, and NS-5% and initial components such as APE and SSOS for comparison (Figure 1). FT-IR can provide valuable information about the chemical structure and interactions of encapsulated extracts like PC [30,31].

In the FT-IR spectra of F-5%, S-5%, and NS-5%, several peaks characteristic of both APE and SSOS can be observed. Firstly, a broad overlapping band in the range 3000–3700 cm^−1^ can be attributed to the N–H and O–H stretching vibrations of secondary amino groups of APE and hydroxyl groups of SSOS, respectively [32]. Prominent characteristic peaks of APE, namely, at 1639 cm^−1^ (amide I), 1538 cm^−1^ (amide II), and 1397 cm^−1^ (C=N) corresponding to the amide groups of PC appear in the spectra of F-5%, S-5%, and NS-5% [33]. Also, a low-intensity peak at 1718 cm^−1^ attributed to the C=O stretching vibrations of an ester group of SSOS is visible in all the spectra of the obtained samples [34]. Another intensive and overlapping band in the range 950–1180 cm^−1^ [35] is present in the spectra of F-5%, S-5%, and NS-5% and is related to the C–OH bending vibration coming from SSOS and APE. In summary, FT-IR analysis of F-5%, S-5%, and NS-5% showed that prominent peaks that are characteristic of SSOS and APE appear in the spectra of all the samples, confirming that immobilisation of APE in SSOS using various techniques was successful.

### 2.2. Characterisation of Encapsulated A. platensis Extract

#### 2.2.1. Morphology of Encapsulated *A. platensis* Extract

**Figure 2:**
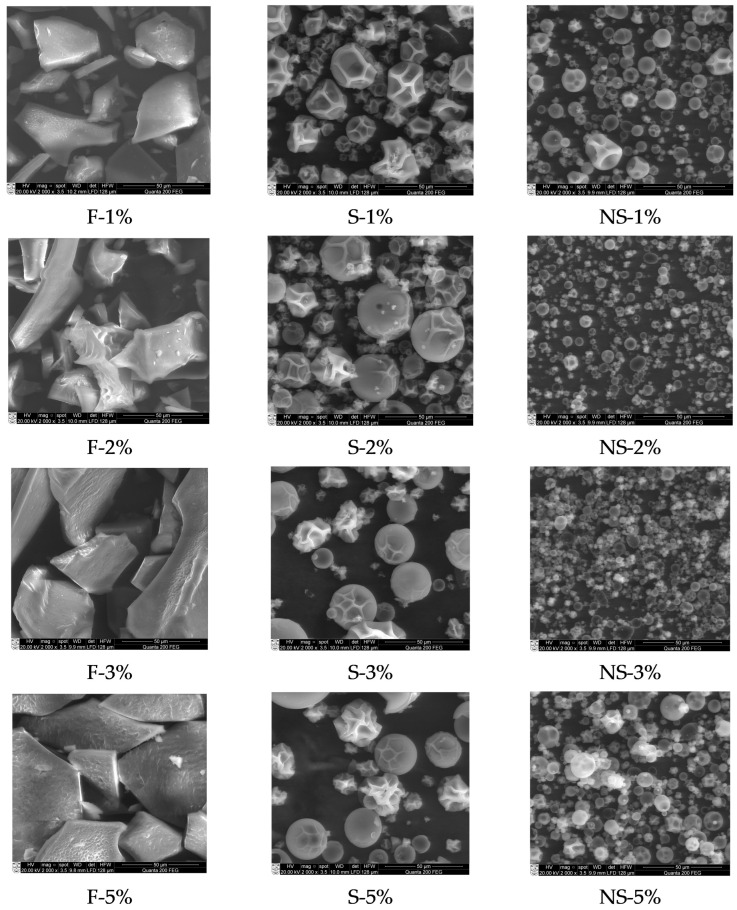
SEM micrographs of freeze-dried (F), spray-dried (S), and nanospray-dried (NS) encapsulated *A. platensis* extract.

Scanning Electron Microscopy (SEM) can provide information about the morphology, particle size distribution, and surface characteristics of the obtained microcapsules. Figure 2 illustrates the SEM images of the encapsulated *A. platensis* extract powders obtained using freeze-drying and spray-drying techniques. When analysing the freeze-dried samples, the structure of the particles resembled shattered glass fragments of varied sizes, as observed in other studies using the same technique [24].

The SEM images of spray-dried and nanospray-dried encapsulated *A. platensis* extract samples showed that the particles had a spherical form with some wrinkled structure. The appearance of the wrinkles on the surface of the capsules was affected by the speed of liquid evaporation during spray-drying. It should be noted that this wrinkled structure was more visible on the capsules of the spray-dried samples, which were obtained by using quite a high inlet temperature of 190 °C. Usually, the diameter of the microcapsules falls within the range of 1–1000 µm, and the spheroidal form is often preferred due to its additional processing benefits [36]. In this study, the nanospray-drying technique allowed us to form significantly smaller and uniform microcapsules with a diameter of 2–14 µm compared to the conventional spray-drying method, which yielded slightly larger particles with a diameter of 10–32 µm.

#### 2.2.2. Colour Characteristics of Encapsulated *A. platensis* Extract

**Figure 3:**
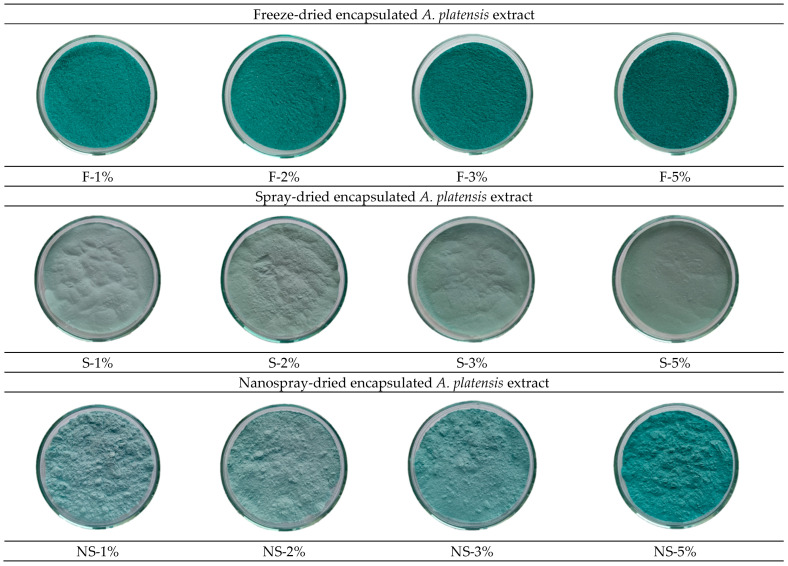
Photographs of freeze-dried (F), spray-dried (S), and nanospray-dried (NS) encapsulated *A. platensis* extract samples.

In this part of the research, the colour of freeze-dried, spray-dried, and nanospray-dried encapsulated *A. platensis* extract was analysed visually as well as using a colourimeter. As can be seen from the photographs, the different encapsulating methods affected the colour of the samples (Figure 3). As obviously indicated from the pictures, blue was not the only coloured phycocyanin pigment present in the PC extract. It could be presumed that during the freeze–thawing cycles of the PC preparation, the disruption of the cells occurred and some chlorophyll might be still present in the extract, giving a green hue [37,38].

Therefore, the impact of different drying methods on the colour parameters (L*, a*, b*) of encapsulated *A. platensis* extract was determined. As can be seen in Table 3, L* a* and b* values of the encapsulated *A. platensis* extract significantly (*p* < 0.05) depended on the technique used. Additionally, colour differences were observed when different amounts of *A. platensis* extract were used for the encapsulation. The L* value indicates a spectrum ranging from black (0) to white (100). The L* value of the freeze-dried, spray-dried, and nanospray-dried samples decreased from 61.80 to 44.60, from 75.48 to 59.01, and from 77.88 to 60.02, respectively, when the content of APE was increased from 1 to 5%, in the initial formulation.

Meanwhile, no significant changes in the value of a* (+a* represents red, −a* represents green) were determined for freeze-dried and spray-dried samples when the content of APE was increased from 1 to 5%. On the contrary, the a* values of the nanospray-dried samples changed from −12.17 to −20.87 with an increasing amount of APE, reflecting the deeper green colour of the samples. Spray-dried samples appeared to be less green than respective freeze-dried and nanospray-dried samples. The values of b* (+b* represents yellow, -b* represents blue) of nanospray-dried and freeze-dried samples varied from −5.54 to −9.03 and from −4.47 to −5.36, respectively, showing deepening blue colour of the samples. However, the values of b* of spray-dried samples, as the amount of APE increased, became more positive and ranged from −2.88 to −0.16, proving the blue colour reduction. The determined values correlate well with the images in Figure 3, where spray-dried samples appear green, though some yellowing could be observed. Comparing the a* and b* values when different encapsulation techniques were used, the most extensive disappearance of greenish-blue colour was determined for spray-dried samples. Previous reports have also shown that PC is sensitive to heat at temperatures over 60 °C, leading to PC reduction and possibly forming the conjugates with subsequent colour changes [29,39,40]. It can be concluded that the significant change in the intensity of the blue-green colour of the spray-dried samples compared to freeze-dried and nanospray-dried samples can be related to the degradation of PC during the spray-drying process at high temperatures. Thus, *A. platensis* extract was better protected during encapsulation through freeze-drying and nanospray-drying techniques, and it is evident that spray-drying is less suitable for encapsulating algae.

#### 2.2.3. Thermogravimetric (TG) Analysis

**Figure 4:**
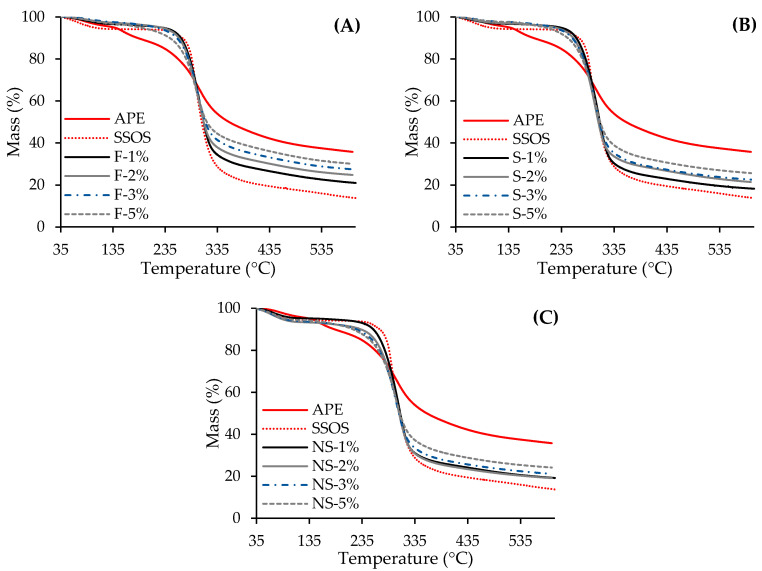
TGA curves of *A. platensis* extract (APE), starch sodium octenyl succinate (SSOS), and freeze-dried (**A**), spray-dried (**B**), and nanospray-dried (**C**) samples with various encapsulated amounts of APE in a nitrogen atmosphere at a heating rate of 10 °C/min.

Thermal stability is an important parameter when evaluating encapsulated samples, especially when using APE, which is relatively thermally unstable [41]. TG analysis was used to investigate the thermal degradation of freeze-dried, spray-dried, and nanospray-dried samples with various encapsulated APE amounts. The thermograms recorded in an inert nitrogen atmosphere are presented in Figure 4. Table 4 presents the relevant characteristic parameters of the thermal decomposition of all samples.

As can be observed from the data presented in Figure 4, three main steps can be discerned in thermograms of all the samples except for SSOS, which only has 2 steps. The initial mass loss step from 35 °C to about 110 °C was related to the loss of bound or adsorbed moisture, which was noticeable for all the samples. During this step, the mass loss of different samples varied between 2.2–6.7%. Samples obtained using the nanospray-drying technique had almost twice the amount of water (4.8–6.7%) compared to samples obtained through spray-drying and freeze-drying (2.2–3.2%).

The second step in the temperature range from about 110 to 180 °C, occurring due to the decomposition of volatiles of APE [42], was most noticeable in the thermogram of APE, with a mass loss of about 5.6% and onset decomposition temperature (*T_o_*) of 140 °C. Meanwhile, the second step was less pronounced in the thermograms of samples obtained using different techniques, with mass losses varying from 0.5% to 1.9%. As the amount of encapsulated APE in the samples increased for different acquisition techniques, mass losses of this step also increased, indicating that encapsulation of different amounts of APE was successful. The onset decomposition temperature (*T_o_*) of this step of samples was higher compared to APE: For spray-dried samples with various amounts of APE encapsulated, it was in the range of 148–151 °C. For nanospray-dried samples—from 151 to 154 °C, and freeze-dried samples—from 162 °C to 163 °C. These results indicate that encapsulation of APE into SSOS through spray-drying, nanospray-drying, and freeze-drying techniques efficiently increases thermal decomposition temperature.

The most considerable mass losses, around 54.4–80.6% in all the thermograms (Figure 4 and Table 4), were observed during the third step, ranging from 180 °C to 600 °C. During this step, pyrolysis of protein, carbohydrates, lipids and other organic matter occured [43]. The onset decomposition temperature of spray-dried, nanospray-dried, and freeze-dried samples with various amounts of APE encapsulated varied between 259 and 267 °C. Meanwhile, for APE, it was slightly lower at −257 °C; for SSOS, it was higher at −278 °C. Also, during the third step, the values of *T_0_* and the mass loss (Δ*w*) for the same sample acquisition technique slightly decreased as the amount of encapsulated APE increased. Such results showed that encapsulation and the amount of encapsulated APE affected the decomposition profile during the third step. Ash residue at the end of the experiment (Table 4) for the same sample acquisition technique increased as the amount of encapsulated APE increased. This increase correlated to the fact that the ash residue of APE was almost 2.6 times higher than SSOS. High residual char of about 40% of *A. platensis* biomass was observed in other studies [42,44], where high ash levels were linked to inorganic salt content in the biomass.

To summarize TG analysis results, the profile changes of thermograms of freeze-dried, spray-dried, and nanospray-dried samples with various encapsulated APE amounts as compared to neat APE and SSOS confirmed that encapsulation was successful and that all three encapsulation techniques helped to increase the thermal stability of APE. Decomposition temperature increased by 8–11 °C for spray-dried, 11–15 °C for nanospray-dried, and 22–23 °C for freeze-dried samples. However, it should be noted that based on the thermal behaviour of samples, the spray-drying technique provided the least protection to APE against thermal action.

### 2.3. PC Release and In Vitro Antioxidant Activity Assessment

**Figure 5:**
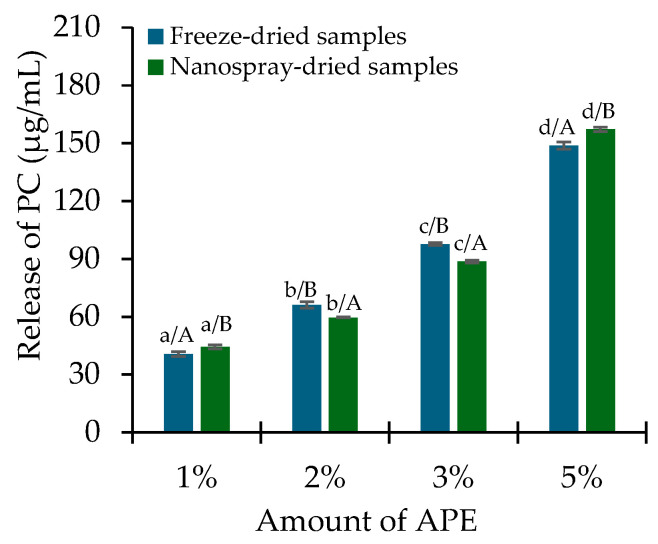
The release of PC from freeze-dried and nanospray-dried samples into saliva simulant solution. a–d: the different lowercase letters within the amount of APE for samples obtained by using the same technique show that the results are significantly different (*p* < 0.05; Duncan test); A,B: the different uppercase letters within the same concentration of APE and different techniques used show that the results are significantly different (*p* < 0.05; Duncan test).

As mentioned before, spirulina extracts and APE capsules have been found to have multiple applications in food and medical applications. Considering the potential application of obtained encapsulated samples as active components in orally disintegrating films, and the lack of information within this field, studies of PC release from the encapsulated *A. platensis* extract into artificial saliva and subsequent bioactivity studies of released APE were performed. Since the efficiency of conventional spray-drying to encapsulate or stabilize PC was low, as discussed in previous sections, we decided to exclude these samples from further analysis. As can be seen from the data in Figure 5, the amount of released PC in artificial saliva depended on the encapsulation technique. As expected, PC release from freeze-dried and nanospray-dried samples into saliva medium increased, ranging from 40.6 to 148.8 µg/mL and 44.3 to 157.2 µg/mL, respectively, with increasing APE load. The differences in the release of PC between samples obtained using different techniques can be explained by considering different PC encapsulation efficiency results (Table 2), where EE values varied between 67.1 and 71.2% for freeze-dried samples and 70.2 and 78.3% for nanospray-dried samples. Thus, a lower encapsulation efficiency determined a lower total PC content in the samples and, consequently, lower amounts of PCs detected in the release medium. This decrease translated to an amount of released PC (%), estimated considering encapsulation extent, that varied from 86.4 to 94.5% and 79.2 to 98.6% for freeze-dried and nanospray-dried samples, respectively. Nevertheless, these results indicated that PC and the APE can be successfully released and remain relatively stable within the tested timeframe in an artificial saliva medium, overall revealing that PC release could be forecasted by choosing the formulation’s appropriate encapsulation technique and composition.

**Figure 6:**
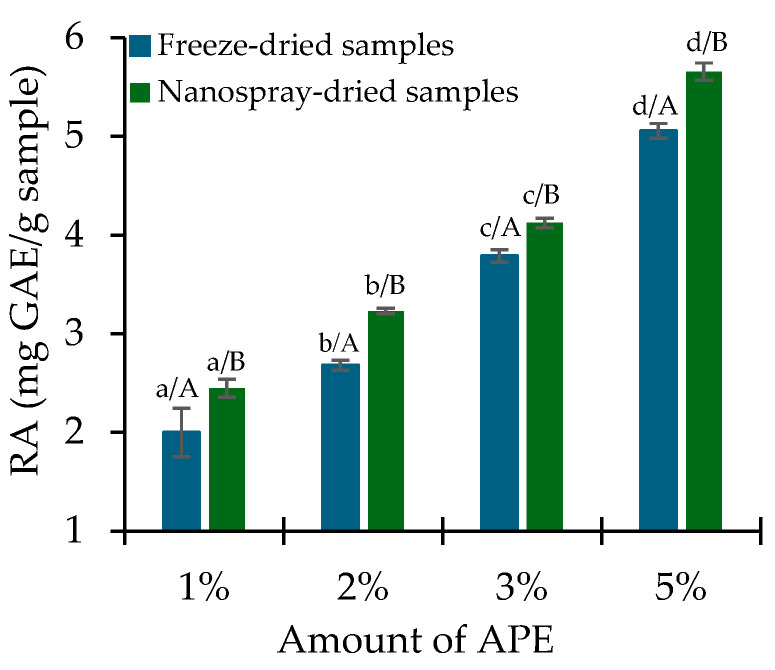
The dependence of reducing activity (RA) measured by Folin–Ciocalteu’s method in saliva simulant on the amount of *A. platensis* extract in the primary formulations and encapsulation technique used. a–d: the different lowercase letters within the amount of APE for samples obtained by using the same technique show that the results are significantly different (*p* < 0.05; Duncan test); A,B: the different uppercase letters within the same concentration of APE and different techniques used show that the results are significantly different (*p* < 0.05; Duncan test).

There is a wealth of evidence supporting PC’s and APE’s antioxidant properties, which have been partially used to explain their anti-inflammatory effects [45]. In fact, studies have evidenced that antioxidant activities are directly related to PC and other bioactive compounds [46]. Reactive oxygen species play a role in many significant medical pathological processes, including inflammatory disorders like periodontitis [47]. Interestingly, both PC and APE are seen as potential treatments for periodontitis [12,28,48]. Therefore, in this part of the study, we assessed the in vitro antioxidant capacity of the obtained capsules in artificial saliva medium via the Folin–Ciocalteu’s, CUPRAC, and ABTS assays. The dependence of the reducing activity (RA) of the samples by Folin–Ciocalteu’s method on the amount of *A. platensis* extract and different encapsulation techniques used is presented in Figure 6. The reducing activity was expressed as mg of gallic acid equivalent (GAE) per gram of dry sample. As can be seen, the RA value significantly (*p* < 0.05) increased by increasing the APE amount in all formulations. A significantly higher amount of RA (*p* < 0.05) was detected for nanospray-dried samples. The saliva simulant solution values ranged from 2.5 to 5.7 (mg GAE/g sample). Meanwhile, freeze-dried samples had lower reducing activity values from 2.0 to 5.1 (mg GAE/g sample).

**Figure 7:**
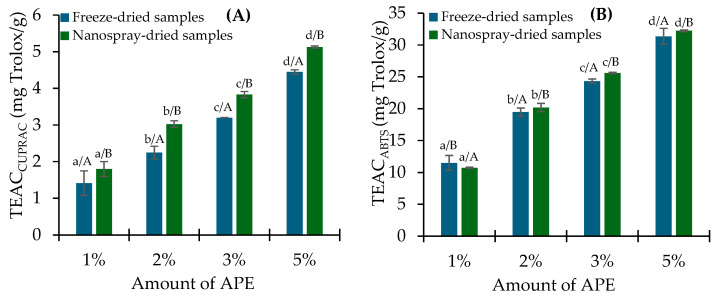
The dependence of antioxidant activity on the amount of *A. platensis* extract in the primary formulations and encapsulation technique used. (**A**) CUPRAC method; (**B**) ABTS method. a–d: the different lowercase letters within the amount of APE for samples obtained by using same technique show that the results are significantly different (*p* < 0.05; Duncan test); A–B: the different uppercase letters within the same concentration of APE and different techniques used show that the results are significantly different (*p* < 0.05; Duncan test).

For CUPRAC and ABTS assays, the antioxidant activity was expressed as mg Trolox per gram of dry sample. Significant differences (*p* < 0.05) were found in the values of Trolox Equivalent Antioxidant Capacity (TEAC) when different amount of APE was encapsulated using different encapsulation techniques (Figure 7). For instance, significantly higher TEAC values (mg Trolox/g) compared to freeze-dried samples were observed for nanospray-dried samples and varied from 1.8 to 5.1 (CUPRAC) (Figure 7A). Meanwhile, TEAC values (mg Trolox/g) for freeze-dried samples were 1.4–4.4 (CUPRAC). Similarly, TEAC values (mg Trolox/g) were increased with increasing APE amount in formulations from 10.7 to 32.2 and from 11.5 to 31.4 (ABTS) for nanospray-dried and freeze-dried samples, respectively (Figure 7B). Calculation of Pearson’s correlation coefficients showed a significant and strong positive correlation between PC release and RA (r = 0.9700, *p* < 0.0001), as well as with antioxidant capacity measured as TEAC_ABTS_ (r = 0.9470, *p* = 0.0004) and TEAC_CUPRAC_ (r = 0.9396, *p* = 0.0005). Additionally, RA exhibited strong correlations with both TEAC_ABTS_ (r = 0.9612, *p* = 0.0001) and TEAC_CUPRAC_ (r = 0.9925, *p* < 0.0001), while TEAC_ABTS_ and TEAC_CUPRAC_ were also highly correlated (r = 0.9662, *p* < 0.0001). These findings indicate that PC is a major contributor to the observed in vitro antioxidant capacity. The encapsulated formulations retain the desired antioxidant capacity upon release. Subsequently, bioactivity studies show that the nanospray-drying technique is more suitable for encapsulating *A. platensis* extract than the freeze-drying method towards potential oral applications.

## 3. Materials and Methods

### 3.1. Materials

Starch sodium octenyl succinate was purchased from Ingredion GmbH (Hamburg, Germany). *A. platensis* dried biomass powder was purchased from UAB Mosus (Vilnius, Lithuania). Ethanol was received from AB Vilniaus degtine (Vilnius, Lithuania). Trolox (6-hydroxy-2,5,7,8-tetramethylchroman-2-carboxylic acid), 2,2′-Azino-bis(3-ethylbenzothiazoline-6-sulfonic acid) diammonium salt, Folin–Ciocalteu’s reagent, neocuproine, copper chloride, and ammonium acetate were purchased from Sigma-Aldrich (St. Louis, MO, USA). Sodium carbonate, sodium chloride, potassium chloride, potassium persulfate, potassium dihydrogen phosphate, disodium phosphate, potassium thiocyanate, urea, sodium sulfate, ammonium chloride, calcium chloride dihydrate, and sodium hydrogen carbonate were obtained from UAB Eurochemicals (Vilnius, Lithuania).

### 3.2. Preparation of A. platensis Extract

*A. platensis* extract was obtained using the freezing–thawing method with some modifications [49]. In short, 30 g of Spirulina dried biomass was suspended in 1 L of distilled water, and the obtained slurry was mixed in the dark at 500 rpm for 45 min using a magnetic stirrer. PCs were extracted by applying 3 cycles of repeated freezing (−20 °C, 12 h) and thawing (~25 °C, 12 h). Cell debris was removed by centrifugation at 8000 rpm for 5 min, and the supernatant was filtered through a 100 µm glass filter. The obtained filtrate was frozen and freeze-dried using SP Scientific Freeze Dryer (SP Industries, Warminster, PA, USA). Prepared *A. platensis* powder was denominated as APE and stored in the freezer (−20 °C) until further use.

The concentration of PC in the extract solution was determined spectrophotometrically using the following equation:

(1) $$PC\;(\frac{mg}{ml})=\frac{A_{620}-0.474\times A_{652\;}}{5.34}$$

where A_620_ is the absorbance of the sample solution at 620 nm, and A_652_ is the absorbance of the sample solution at 652 nm.

The total PC content expressed on a dry weight basis (mg/g) was calculated using the following equation:

(2) $$PC\;content\;\left(\frac{mg}{g}\right)=\frac{phycocyanin\;\left(\frac{mg}{ml}\right)\times volume\;of\;solvent\;\left(ml\right)}{\;weight\;of\;sample\;\left(g\right)}$$

### 3.3. Characterisation of the Aqueous A. platensis Extract

The proximate composition of the obtained extract was assessed following previously reported procedures. Briefly, the total carbohydrate content was determined using the sulfuric acid–UV method, as reported by Albalasmeh et al. [50]. The protein content was obtained following the AOAC, Method 960.52, Microchemical Determination of Nitrogen-Micro-Kjeldahl Method. The moisture and ash contents determined gravimetrically according to the AOAC standard method, whereas the lipid content was assessed gravimetrically following extraction with n-hexane [51].

Moreover, the amino acid profile was analysed chromatographically as previously reported with slight modifications [52]. Analyses were performed in a Shimadzu LC-2050C3D UPLC system connected to a Shimadzu 8045-mass spectrometer (Shimadzu Corporation, Kyoto, Japan) using a Discovery^®^ HS F5 (15 cm × 2.1 mm, 3 µm) column (Merck Group, Darmstadt, Germany).

### 3.4. Preparation of Dispersions Containing A. platensis Extract

The aqueous formulations containing 10% (*w*/*w*) of starch sodium octenyl succinate (SSOS) and 1, 2, 3, and 5% (*w*/*w*) of *A. platensis* extract (APE) were prepared by using rotor-stator homogenizer Ultra-Turrax T25 digital (IKA, Staufen, Germany) at 12,000 rpm for 5 min.

APE to wall material ratio was selected based on the studies of other authors [25,53] and our preliminary studies aiming to yield stable dispersions. The size of the particles in the SSOS-APE aqueous dispersions was assessed according to the cumulative distribution of intensity using a DelsaTM Nano C particle size meter (Beckman Coulter, Malvern, UK), which uses photon correlation spectroscopy to determine particle size by measuring the rate of fluctuations in the laser light intensity scattered by particles. The non-negative least-squares (NNLS) algorithm was used to analyse dynamic light scattering data for the particle size distribution. All scattered light measurements were made at an angle of 165°. For particle size measurements, the samples were diluted with distilled water to reach a concentration of 1% *w*/*v* to avoid multiple scattering effects. Measurements were carried out in triplicate.

### 3.5. Encapsulation of A. platensis Extract Using Freeze-Drying and Spray-Drying Techniques

**Scheme 1:**
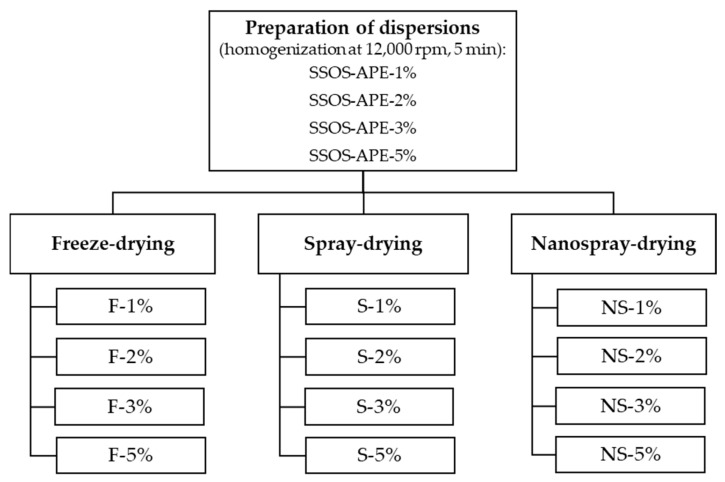
Steps of preparation of starch sodium octenyl (SSOS) encapsulated *A. platensis* extract (APE).

The prepared SSOS-APE aqueous dispersions (Section 3.3) with varying APE content were used to obtain the solid particles with encapsulated extract by employing freeze-drying, conventional and nano-spray-drying (see Scheme 1). The SSOS-APE dispersions were frozen and freeze-dried using SP Scientific Freeze Dryer (SP Industries, Warminster, PA, USA). Furthermore, SSOS-APE formulations were spray dried using Mini Spray Dryer Buchi 190 (Büchi Labortechnik AG, Flawil, Switzerland) under the following conditions: inlet temperature of 190 ± 5 °C, outlet temperature of 110 ± 5 °C, pressure of 39 hPa, aspirator setting of 12, air flow rate of 700 L/h, sample feed rate of 5 mL/min, and nozzle diameter of 0.5 mm.

Also, the formulations were nanospray-dried using Buchi Nano Spray Dryer B-90 (Büchi Labortechnik AG, Flawil, Switzerland) by applying the following parameters: the drying gas (air) flow rate of 98 ± 3 L/min, inlet temperature of 120 ± 1 °C, outlet temperature of 45 ± 5 °C, pressure of 28 ± 2 hPa; the diameter of the spray mesh holes was 7.0 μm. The inlet and outlet temperatures in the case of both spray-drying and nanospray-drying techniques were kept as low as possible to avoid the degradation of the extract and to insure the stable thermal processing of the dispersions.

In this study, obtained samples were named based on the encapsulation technique abbreviation and the amount of *Arthrospira platensis* extract (APE) in the formulation used, e.g., freeze-dried samples with 1% of APE, spray-dried samples with 1% of APE, and nanospray-dried samples with 1% of APE were named as F-1%, S-1%, and NS-1%), respectively.

The encapsulation efficiency *(EE %)* was determined by detecting the concentration of the PC in the initial formulation and the concentration after the encapsulation by re-dispersing and dissolving the obtained powders in distilled water. Encapsulation efficiency (*EE %)* was calculated using the following formula:

(3) $$EE\;\left(\%\right)=\frac{W_{t}}{W_{i}}\times 100$$

where *W_t_* is the total amount of PC after encapsulation (mg/g) and *W_i_* is the theoretical total amount of PC in the initial formulations (mg/g).

The maximal theoretical amount of PC was 6.4, 11.7, 16.2, and 23.3 mg/g using 1, 2, 3, and 5% of *A. platensis* extract (*w*/*w*) in initial formulations, respectively.

### 3.6. Analyses of Encapsulated A. platensis Extract Powder

#### 3.6.1. SEM Analyses

Scanning electron microscopy (SEM) images of freeze-dried (F), spray-dried (S), and nano-spray-dried (NS) encapsulated APE powders were obtained using a Quanta 200 FEG scanning electron microscope (FEI, Brno, Czech Republic). Micrographs were taken at a magnification of 2000×.

#### 3.6.2. Colour Measurements

The colour of the freeze-dried (F), spray-dried (S), and nano-spray-dried (NS) encapsulated APE powders was assessed with a colourimeter (Konica Minolta CM-5, illuminant D_65_). The colour characteristics were expressed by CIE system parameters in terms of lightness L* (from 0 (black) to 100 (white)) and chromaticity parameters a* (green (–)/red (+) balance) and b* (blue (–)/yellow (+) balance).

#### 3.6.3. FT-IR Spectroscopic Analysis

FT-IR spectra of the samples were recorded using a Frontier (Perkin–Elmer, Waltham, MA, USA) spectrophotometer with a single reflectance horizontal attenuated total reflectance cell equipped with a diamond crystal. The data were recorded in the spectral range from 650 to 4000 cm^−1^ by accumulating 10 scans with a resolution of 4 cm^−1^.

#### 3.6.4. Thermogravimetric Analysis

Thermogravimetric (TG) analysis was performed using TGA 400 (Perkin–Elmer, Waltham, MA, USA). The measurements were conducted under nitrogen at a flow rate of approximately 20 cm^3^/min, using a typical heating ramp of 10 °C/min from 35 °C to 600 °C. The ceramic pan was filled with about 10 mg of the sample. Pyris Data Analysis software (v.10.1.0.0411) (Perkin–Elmer, Waltham, MA, USA) collected relevant data on thermal decomposition stages.

#### 3.6.5. Release Studies in Artificial Saliva

Artificial saliva was prepared, as described by Pistone et al. [54]. Briefly, 125.6 mg of NaCl, 963.9 mg of KCl, 189.2 mg of KSCN, 654.5 mg of KH_2_PO_4_, 200 mg of urea, 336.5 mg of Na_2_SO_4_, 178 mg of NH_4_Cl, 227.8 mg of CaCl_2_·2H_2_O, and 630.8 mg of NaHCO_3_ were dissolved in 1 L of distilled water. The pH of the medium was 6.8 ± 0.1.

0.1 g of freeze-dried or nanospray-dried sample was dispersed in 10 mL of prepared saliva simulant medium and stirred at 300 rpm for 30 min. Then, the samples were centrifuged at 6000 rpm for 10 min, filtered through the nylon filter (0.45 µm), and used to determine the PC content, total phenolic content, and antioxidant activity.

#### 3.6.6. In Vitro Antioxidant Activity Assessment

The in vitro antioxidant capacity of the samples was assessed by the Folin–Ciocalteu’s, cupric reducing antioxidant capacity (CUPRAC), and 2,2′-azinobis-(3-ethylbenzothiazoline-6-sulfonic acid) (*ABTS^•+^*) scavenging assays as previously described [55,56,57].

Folin-Ciocalteu’s assay. Briefly, 150 µL of sample or blank solution was mixed with 750 μL of Folin-Ciocalteu reagent (diluted with distilled water at 1:9, *v*/*v*). After 3 min, 600 μL of 7.5% NaCO_3_ solution was added. The samples were incubated for 2 h at room temperature, and the absorbance at 760 nm against a reagent blank was measured using a UV-Vis spectrophotometer T60 (PG Instruments, Wibtoft, Great Britain). Reducing activity (RA) was expressed as gallic acid equivalents (mg GAE/g sample), employing a gallic acid calibration curve and linear equation y = 40.367x − 0.0036 with correlation coefficient R^2^ = 0.9988.

CUPRAC assay. CUPRAC assay was performed by mixing 400 μL of a sample (10 mg/mL) or blank with 400 μL of each buffer solution: ammonium acetate, neocuproine, and copper chloride. Vials were kept in the dark for 30 min. Subsequently, the absorbance was measured at 450 nm using UV-Vis spectrophotometer T60 (PG Instruments, Wibtoft, Great Britain), and the antioxidant capacity was expressed as Trolox equivalent antioxidant capacity TEAC_CUPRAC_ (mg Trolox/g) using a calibration curve and linear equation y = 0.004x + 0.0155 with R^2^ = 0.9997.

*ABTS^•+^* assay. First, a stock solution of ABTS^•+^ was prepared by dissolving 0.0549 g of ABTS and 0.0038 g of K_2_S_2_O_8_ in 50 mL of distilled water. The stock mixture was stored in the dark at room temperature for 16 h before use. Phosphate buffer solution (PBS) was prepared as follows: 4.09 g of NaCl, 0.135 g of KH_2_PO_4_, 0.71 g of Na_2_HPO_4_, and 0.075 g of KCl were dissolved in 500 mL of distilled water. The working radical solution was prepared by diluting the stock solution with PBS until an absorbance of AU 0.700 at 734 nm absorbance was reached. Then, 25 μL of sample (10 mg/mL) or blank was mixed with 1500 μL of working radical solution and kept in the dark for 2 h. Then, the absorbance was measured at a wavelength of 734 nm. The antioxidant capacity was expressed as Trolox equivalent antioxidant capacity TEAC_ABTS_ (mg Trolox/g) using a calibration curve and linear equation y = −0.0004x + 0.6691 with R^2^ = 0.9982.

### 3.7. Statistical Analysis

The data were statistically processed using a one-way analysis of variance (ANOVA for Excel, version 2.2). Except when noted otherwise, all analyses were conducted in triplicate, and the outcomes were presented as mean values ± standard deviations. Duncan’s multiple range test was utilised to determine significant differences among the characteristic parameter values at a probability level of *p* < 0.05. GraphPad Prism 10.4.1 software was used to calculate Pearson’s correlation coefficients (two-tailed *p* < 0.05) between PC release, RA, TEAC_CUPRAC_ and TEAC_ABTS_ values in artificial saliva medium.

## 4. Conclusions

*A. platensis* extract was successfully encapsulated by freeze-drying and two spray-drying techniques, conventional and nanospray-drying, to obtain microparticles by using starch sodium octenyl succinate as wall material. The encapsulation efficiency of 67–71% was characteristic of freeze-dried samples; for nanospray-dried samples it ranged from 70 to 78%, whereas significantly lower values (from 38 to 42%) were determined for spray-dried samples. The significant decrease in the colour of spray-dried samples was also determined. Thus, the higher temperature of spray-drying processing significantly impacted *A. platensis* extract stability during the encapsulation. Nevertheless, thermogravimetric analysis demonstrated that encapsulation, in general, enhanced the thermal stability of *A. platensis* in all samples compared to the crude extract. The results indicated that the nano-spray-drying technique was the most effective and allowed the achievement of the smallest particles with good colour and thermal stability, antioxidant capacity, and demonstrated the potential of this technique when producing algae-based materials for nutraceutical or pharmaceutical products such as periodontal films. As *A. platensis* appears to be a promising bioactive agent with a wide range of antibacterial, antioxidant, and anti-inflammatory properties, its controlled delivery within periodontal pockets could alter the pathogenic flora and probably could diminish periodontitis. Therefore, the further steps of this study will include the testing of the developed materials against oral pathogens such as *Streptococus mutans*, *Porphyromonas gingivalis*, *Aggregatibacter actinomycetemcomitans*, *Candida albicans*, etc. Finally, as the evidence indicate that the fermentation process of *A. platensis* may significantly increase the bioactivity of algae extract [53,58], our further studies will also focus on optimising the fermentation process of *A. platensis* extracts and developing encapsulation strategies for fermented *A. platensis* extracts.

## Figures and Tables

**Table 1:** Characteristics of starch sodium octenyl succinate and *A. platensis* extract dispersions.

Sample *	Solids Content in Dispersion, wt.%		Particle Size (nm)	PI
	SSOS	APE		
SSOS-APE-1%	10	1	396 ± 21 ^a^	0.32 ± 0.01 ^ab^
SSOS-APE-2%	10	2	423 ± 25 ^ab^	0.34 ± 0.05 ^ab^
SSOS-APE-3%	10	3	466 ± 23 ^b^	0.34 ± 0.03 ^b^
SSOS-APE-5%	10	5	542 ± 20 ^c^	0.34 ± 0.04 ^ab^

* Starch sodium octenyl succinate (SSOS) and *A. platensis* extract (APE) dispersions containing 1, 2, 3, and 5% of APE. ^a–c^: the different letters within the same column show that the results are significantly different (*p* < 0.05; Duncan test).

**Table 2:** Total content of PC in solid particles and encapsulation efficiency of PC.

Sample *	Total Content of PC (mg/g)	Encapsulation Efficiency of PC (%)
F-1%	4.3 ± 0.1 ^a/C^	67.1 ± 1.0 ^abc/C^
F-2%	7.4 ± 0.0 ^b/C^	63.6 ± 0.1 ^a/C^
F-3%	11.3 ± 0.2 ^c/C^	69.8 ± 1.1 ^bc/C^
F-5%	16.6 ± 0.4 ^d/B^	71.2 ± 1.8 ^c/B^
S-1%	2.4 ± 0.2 ^a/A^	38.4 ± 3.8 ^ab/A^
S-2%	5.1 ± 0.2 ^b/A^	43.9 ± 1.4 ^a/A^
S-3%	6.3 ± 0.0 ^c/A^	39.8 ± 0.2 ^ab/A^
S-5%	9.7 ± 0.1 ^d/A^	41.6 ± 0.5 ^ab/A^
NS-1%	4.5 ± 0.1 ^a/C^	70.2 ± 2.1 ^b/C^
NS-2%	6.7 ± 0.1 ^b/C^	57.8 ± 0.6 ^a/C^
NS-3%	11.2 ± 0.0 ^c/C^	69.0 ± 0.1 ^b/C^
NS-5%	18.3 ± 0.0 ^d/C^	78.3 ± 0.0 ^c/C^

* F—freeze-dried samples; S—spray-dried samples; NS—nanospray-dried samples; ^a–d^: the different lowercase letters within the column for samples obtained by using the same technique show that the results are significantly different (*p* < 0.05; Duncan test); ^A–C^: the different uppercase letters within the column for the same concentration of APE and different technique used to show that the results are significantly different (*p* < 0.05; Duncan test).

**Table 3:** The colour parameters of encapsulated *A. platensis* extract.

Sample *	Colour Parameters		
	L*	a*	b*
F-1%	61.80 ± 0.36 ^d/A^	−16.93 ± 0.17 ^a/A^	−5.18 ± 0.05 ^b/B^
F-2%	55.37 ± 0.10 ^c/A^	−15.36 ± 0.12 ^c/A^	−4.47 ± 0.03 ^c/A^
F-3%	51.48 ± 0.29 ^b/A^	−16.22 ± 0.04 ^b/A^	−5.19 ± 0.02 ^b/B^
F-5%	44.60 ± 0.35 ^a/A^	−16.17 ± 0.09 ^b/B^	−5.36 ± 0.10 ^a/B^
S-1%	75.48 ± 0.43 ^d/B^	−10.53 ± 0.08 ^a/C^	−2.88 ± 0.08 ^a/C^
S-2%	69.59 ± 0.21 ^c/B^	−10.24 ± 0.05 ^b/C^	−1.50 ± 0.02 ^b/B^
S-3%	63.95 ± 0.26 ^b/B^	−10.01 ± 0.09 ^c/C^	−1.22 ± 0.02 ^c/C^
S-5%	59.01 ± 0.35 ^a/B^	−9.47 ± 0.06 ^d/C^	−0.16 ± 0.03 ^d/C^
NS-1%	77.88 ± 0.54 ^d/C^	−12.17 ± 0.03 ^c/B^	−5.54 ± 0.07 ^c/A^
NS-2%	74.89 ± 0.01 ^c/C^	−12.09 ± 0.22 ^c/B^	−4.65 ± 0.16 ^d/A^
NS-3%	72.17 ± 0.09 ^b/C^	−15.14 ± 0.08 ^d/B^	−6.78 ± 0.09 ^b/A^
NS-5%	60.02 ± 0.01 ^a/C^	−20.87 ± 0.17 ^a/A^	−9.03 ± 0.04 ^a/A^

* F—freeze-dried samples; S—spray-dried samples; NS—nanospray-dried samples; ^a–d^: the different lowercase letters within the column for samples obtained by using the same technique show that the results are significantly different (*p* < 0.05; Duncan test); ^A–C^: the different uppercase letters within the column for the same concentration of APE and different technique used to show that the results are significantly different (*p* < 0.05; Duncan test).

**Table 4:** Characteristic parameters of thermal degradation of APE, SSOS, and freeze-dried, spray-dried, and nanospray-dried samples with various amounts of APE encapsulated.

Sample *	I Step	II Step		III Step			Ash Residue at 600 °C (%)
	Δ*w* (%)	*T_o_* (°C)	Δ*w* (%)	*T_o_* (°C)	Δ*w* (%)	*T_max_* (°C)	
APE	4.3 ± 0.3	140.1 ± 0.2	5.6 ± 0.2	257.3 ± 0.3	54.4 ± 0.2	299.0 ± 0.2	35.8 ± 0.3
SSOS	5.7 ± 0.1	–	–	278.2 ± 0.2	80.6 ± 0.1	297.1 ± 0.2	13.8 ± 0.1
F-1%	3.2 ± 0.3	163.4 ± 0.2	0.8 ± 0.2	267.2 ± 0.1	75.1 ± 0.3	300.1 ± 0.1	20.9 ± 0.3
F-2%	2.5 ± 0.3	162.0 ± 0.1	1.0 ± 0.1	268.1 ± 0.2	71.7 ± 0.1	297.0 ± 0.2	24.8 ± 0.2
F-3%	2.2 ± 0.2	163.3 ± 0.1	1.3 ± 0.1	266.0 ± 0.1	69.1 ± 0.1	296.2 ± 0.2	27.5 ± 0.1
F-5%	2.8 ± 0.2	162.1 ± 0.3	1.9 ± 0.1	261.0 ± 0.2	65.2 ± 0.2	294.1 ± 0.1	30.1 ± 0.2
S-1%	3.2 ± 0.2	148.0 ± 0.1	0.5 ± 0.2	269.0 ± 0.2	78.1 ± 0.1	303.1 ± 0.2	18.2 ± 0.3
S-2%	2.9 ± 0.3	149.3 ± 0.3	0.8 ± 0.1	264.3 ± 0.2	74.9 ± 0.2	298.0 ± 0.1	21.0 ± 0.1
S-3%	2.2 ± 0.3	150.1 ± 0.2	1.1 ± 0.2	264.1 ± 0.1	74.1 ± 0.3	298.3 ± 0.2	22.6 ± 0.2
S-5%	2.3 ± 0.1	151.3 ± 0.2	1.5 ± 0.1	261.1 ± 0.1	70.6 ± 0.2	296.3 ± 0.1	25.6 ± 0.2
NS-1%	4.8 ± 0.3	154.4 ± 0.3	0.6 ± 0.2	267.2 ± 0.2	75.5 ± 0.2	303.0 ± 0.1	19.2 ± 0.1
NS-2%	6.7 ± 0.3	153.1 ± 0.2	1.0 ± 0.1	265.3 ± 0.2	73.2 ± 0.3	299.2 ± 0.1	19.1 ± 0.2
NS-3%	6.3 ± 0.2	155.1 ± 0.3	1.2 ± 0.2	260.0 ± 0.1	71.3 ± 0.3	298.1 ± 0.2	21.2 ± 0.3
NS-5%	5.7 ± 0.3	151.3 ± 0.4	1.7 ± 0.3	259.2 ± 0.2	68.4 ± 0.2	296.3 ± 0.1	24.1 ± 0.1

* APE—*A. platensis* extract; SSOS—starch sodium octenyl succinate; F—freeze-dried samples; S—spray-dried samples; NS—nanospray-dried samples.

## Data Availability

Data will be made available upon request.

## Abbreviations

The following abbreviations are used in this manuscript:

PC	Phycocyanin
EE	Encapsulation efficiency
SSOS	Starch sodium octenyl succinate
APE	*Arthrospira platensis* extract
TEAC	Trolox equivalent antioxidant capacity
TGA	Thermogravimetric analysis
FT-IR	Fourier Transform Infrared Spectroscopy
SEM	Scanning Electron Microscopy
